# Evaluation of the acceptability, feasibility and effectiveness of two methods of involving patients with disability in developing clinical guidelines: study protocol of a randomized pragmatic pilot trial

**DOI:** 10.1186/1745-6215-15-118

**Published:** 2014-04-10

**Authors:** Marie-Eve Lamontagne, Kadija Perreault, Marie-Pierre Gagnon

**Affiliations:** 1 Center for Interdisciplinary Research in Rehabilitation and Social Integration, Institut de réadaptation en déficience physique de Québec, 525, boulevard Wilfrid-Hamel, Québec G1M 2S8, QC, Canada; 2 Département de réadaptation, Faculté de Médecine, Université Laval, 1050, avenue de la Médecine, Québec G1V 0A6, QC, Canada; 3 Faculté des sciences infirmières, Université Laval, 1050, avenue de la Médecine, Québec G1V 0A6, QC, Canada; 4 Centre hospitalier de Québec Research Center, 10, rue de l'Espinay, D7-722, Québec G1L 3 L5, QC, Canada

**Keywords:** Clinical practice guideline, Brain injury, Patient and population participation, Adaptation, Disabilities

## Abstract

**Background:**

Despite growing interest in the importance of, and challenges associated with the involvement of patient and population (IPP) in the process of developing and adapting clinical practice guidelines (CPGs), there is a lack of knowledge about the best method to use. This is especially problematic in the field of rehabilitation, where individuals with disabilities might face many barriers to their involvement in the guideline development and adaptation process. The goal of this pilot trial is to document the acceptability, feasibility and effectiveness of two methods of involving patients with a disability (traumatic brain injury) in CPG development.

**Methods/Design:**

A single-blind, randomized, crossover pragmatic trial will be performed with 20 patients with traumatic brain injury (TBI). They will be randomized into two groups, and each will try two alternative methods of producing recommendations; a discussion group (control intervention) and a Wiki, a webpage that can be modified by those who have access to it (experimental intervention). The participants will rate the acceptability of the two methods, and feasibility will be assessed using indicators such as the number of participants who accessed and completed the two methods, and the number of support interventions required. Twenty experts, blinded to the method of producing the recommendations, will independently rate the recommendations produced by the participants for clarity, accuracy, appropriateness and usefulness.

**Discussion:**

Our trial will allow for the use of optimal IPP methods in a larger project of adapting guidelines for the rehabilitation of individuals with TBI. Ultimately the results will inform the science of CPG development and contribute to the growing knowledge about IPP in rehabilitation settings.

**Trial registration:**

Clinical trial KT Canada 87776.

## Background

Clinical practice guidelines (CPGs) are systematically developed statements designed to help practitioners and patients decide on health care for specific clinical circumstances [1]. CPGs are expected to improve the quality of care and patient outcomes [2], and they are an important pillar of evidence-based practice [3].

The development and/or adaptation of CPGs relies on a rigorous process encompassing a search for scientific evidence and its appraisal by experts such as researchers, clinicians, managers and decision-makers and, importantly, by patients [4,5]. During subsequent consensus meetings, the experts produce recommendations for care based on the scientific evidence and their experience [3]. In addition to scientific evidence and clinicians’ experience, best quality CPGs should consider the opinions and preferences of the target patient population [6]. This aspect is often addressed through the involvement of patient and population (IPP) in the process of CPG development. In the general field of medicine, Légaré *et al.*[2] describe three broad types of IPP in CPG development: 1) communication of the CPG to the target patient population (for example guidelines are disseminated to patients that might be concerned or to their associations); 2) consultation with the patient population (via surveys or focus groups or scientific studies designed to get patients’ perspective on a given aspect of care); or 3) direct participation of patients and population in CPG development (such as involvement in consensus meetings). IPP is deemed to be an effective way to incorporate patients’ values, preferences, perspectives and knowledge in CPGs [2]. It is also seen as a way to improve the feasibility of implementation and comprehensiveness of CPGs, to promote patient and public influence over their development, and to adapt CPGs to the target population [2].

Despite growing interest in the importance of, and challenges associated with IPP in the CPG development and adaptation process, there is a lack of knowledge about the best way to involve the patient and population in such activities [2], and suggested frameworks for IPP in this process appear to have little empirical basis [7]. Only a few authors have empirically tested different methods to involve patients and populations in CPG development. Van Wersch and Eccles [8] tested four different methods of IPP in CPG development while working with the North of England evidence-based development program. These methods were: 1) inviting individual patients to participate in multidisciplinary CPG development groups and assigning them the experts’ tasks of appraising evidence and helping to draft recommendations; 2) ‘one-off’ meetings with patients; 3) workshops with patients; and 4) incorporating a consumer advocate in guideline development groups. These authors drew many conclusions from their observations and experience. They showed that when involving individual patients (method 1), patients contributed infrequently to the discussions, had problems with the use of technical language and their contributions were not subsequently acted upon. In a ‘one-off’ meeting experience with patients, the authors found that patients also reported difficulties with understanding medical terminology and jargon, and their understanding of the use of scientific evidence in order to contribute to more cost-effective health care remained unclear. A series of workshops with patients, while relatively resource-intensive, allowed for the explanation of the technical elements of guideline development to patients, who could then engage in this process and make relevant suggestions. A final experiment incorporating a consumer advocate (instead of an actual patient) in guideline development groups was deemed effective because the advocate had previous similar experience and was familiar with the medical terminology. Indeed, he was used to having discussions with health professionals and felt confident speaking in the group. The authors concluded that no method was clearly superior to the others, and that further work was required on how best to get meaningful consumer involvement in CPG development [8]. More recently, Diaz Del Campo *et al.*[9] reported their experience of two methods of IPP, the first being patient consultation (via in-depth interviews and focus groups) and the second being the active participation of patients in all steps in CPG development, such as appraisal of evidence or consensus development meetings. Based on their findings, the authors concluded that patient involvement in CPG development was very helpful to incorporate patients’ views and needs into CPGs, but that it was crucial to have specific support for patients, who must be precisely selected using defined eligibility criteria in order to facilitate an effective engagement [9]. den Breejen *et al.*[10] explored the use of a Wiki tool in the development of CPGs on infertility. A Wiki is a webpage that can be modified by those who have access to it. In this study, the Wiki was built using MediaWiki software, and it was structured according to the draft recommendations of the guidelines. Over the seven months of the study, the Wiki attracted 298 visitors, yielding 289 recommendations that were subsequently rated in a top five or top three for each section of the guidelines. The authors concluded that the Wiki was a promising and feasible tool for patients to participate in guideline development and identify targets for improvement.

The scarcity of knowledge about how to best involve patients and populations in CPG development is problematic in the context of rehabilitation services. In rehabilitation, the adoption of CPG recommendations by clinicians and patients holds the potential to have a permanent and decisive impact on functional status, social participation and quality of life. Rehabilitation services typically focus on function and disabilities, including trying to alleviate chronic conditions that impact an individual’s participation in his or her usual activities. This specific context is one of the situations that strongly call for IPP in CPG development [11]. In addition, because rehabilitation is generally described as a client-centered field [12], not involving patients and populations in defining CPGs for a given condition may appear counterintuitive. To add to the complexity, individuals who receive rehabilitation services often live with a variety of physical, communication-related, cognitive or intellectual disabilities that influence their day-to-day participation. These disabilities may make their active participation in the recommendation development process more challenging, albeit crucial. For example, an individual who suffers from a stroke might have severe aphasia that makes interacting with others more laborious. An individual living with a spinal cord injury might struggle with manipulating papers, including the numerous documents to be considered by the experts in CPG development. Other patients, for example those with traumatic brain injury (TBI), might have multiple disabilities, including cognitive problems that may make understanding scientific evidence very difficult, fatigue-related problems that limit participation in long meetings or physical limitations that make travelling to and accessing consensus meetings difficult.

It is crucial to increase our knowledge of effective ways to involve patients and populations with disabilities in the development of clinical practice guidelines. This is evident for several reasons: the growing popularity of CPGs in clinical reasoning and evidence-based practice, the increasing recognition of IPP in CPGs, the paucity of research in rehabilitation on IPP in CPGs, and the barriers that individuals with disabilities might experience while participating in CPG development in rehabilitation.

The goal of this pilot study is to document the acceptability, feasibility and effectiveness of two methods of involving patients with a disability in CPG development. The population of individuals with TBI was selected for this study, since a large-scale initiative to adapt and implement guidelines is currently in its early phases in Canada and questions were raised about patient participation in the development process. Given the often severe disabilities experienced by individuals with TBI, it is possible that the optimal method could be used with success in other populations often presenting multiple disabilities, such as those living with stroke, spinal cord injury or multiple sclerosis.

## Methods/Design

### Study context and design

This pilot study will be performed in the context of adapting CPGs for patients with TBI, corresponding to the adaptation of recommendations stage of the procedures suggested by the ADAPTE working group to adapt clinical guidelines to local context [5]. A single-blind, randomized, crossover pragmatic trial [13,14] will be performed with patients with TBI. They will try two alternative methods of producing recommendations (described below); a discussion group (control intervention) and a Wiki (experimental intervention). The participants will be asked to discuss two recommendations chosen from the Scottish Intercollegiate Guideline Network (SIGN) guidelines for the rehabilitation of individuals with TBI [1]. This CPG was chosen because it was found to have a high quality score on the Appraisal of Guidelines for Research and Evaluation (AGREE II) tool [15,16]. Two researchers selected the recommendations to be adapted by the participants based on their applicability to all patients with TBI (Additional file 1). As these individuals may have various clinical profiles and trajectories, it is important to choose recommendations that are relevant for all participants. The research protocol was approved by the Research Ethics Committee of the Institut de réadaptation en déficience physique de Québec (English translation: Québec Rehabilitation Institute for Physical Disability) (2013–0348).

### Participants

A convenience sample of 20 patients will be recruited from members of the Québec community-based association of individuals with TBI. These patients must have suffered a moderate-to-severe TBI (Glascow Coma Scale <13), have been living with TBI for two to four years (to ensure they still remember their rehabilitation process), be French-speaking, be able to use a computer, and be able to participate in a two-hour group meeting. A letter will first be sent to association members, informing them about the research project. One week later, a trained employee from the association will contact members by phone to answer any questions and verify their interest in participating in the study. The recruitment calls will be performed in a random manner - the list of members will be randomized with regard to the order of members. The recruitment will continue until 20 potential participants have been recruited. This number is optimal to allow for the participation of an adequate number of individuals in the focus groups (half the sample, n = 10) [17,18], while remaining ethically sound for a pilot trial. The list of participants will then be given to the researchers.

Eligibility will be evaluated by contacting potential participants by phone. Eligible participants will be interviewed to document their sociodemographic and trauma characteristics. They will then be invited to attend a training session to be held one week later. Information about the session will also be mailed and calls will be made two days before to remind participants and to avoid difficulties related to memory problems, which are very prevalent in persons with TBI.

### Procedures

The trial will compare two interventions which are identified as the two methods of IPP, focus group and Wiki. The participants will try each intervention once, in accordance with crossover trial methodology. The trial will take place in the community, at the association’s office (for the training session and discussion group) and at home (Wiki).

### Training

The patients will first be informed about the project and sign the consent form. After agreeing to participate in the study, they will receive in-person training on guidelines and IPP using educational material on the subject developed by the Health Council of Canada [19]. This training is essential to ensure the participants have some knowledge about CPGs and IPP and should be able to participate effectively in the trial. The participants will also be informed about the two interventions to be tested, and will be given a written procedure to support their participation in the trial. The meeting will last approximately one hour.

After the training, participants will be randomized using a random number generator, into Group 1 or Group 2 by a researcher blinded to the particular intervention [20]. The allocation concealment will be performed using the list of participants ordered randomly.

### Intervention 1 (control): discussion group

Group 1 participants will first be invited to participate in a two-hour focus group aiming to adapt the particular recommendation to encompass their values and preferences. Three days before the focus group they will receive by email: 1) instructions about the time and place of the group, 2) the recommendation to be discussed, and 3) a synthesis of the evidence that was used to produce that specific recommendation. The focus group will be moderated by an experienced research professional using certain predetermined issues to discuss, but leaving room for emerging themes. It will begin with a quick reminder about the goal of the study and the goal of the focus group. The synthesis of the evidence that was used to produce the recommendation will be reviewed. Then the participants will be asked to discuss the recommendation. The discussion will aim to get the participants’ opinion about the recommendation, elicit the participants’ preferences about the recommendation, and explore possible changes to make to the recommendation. Support interventions will be provided as needed by the moderator to encourage participants to interact. The results of the discussion will be recorded on flip charts as the discussion proceeds. These visual cues will facilitate the participation of users who have cognitive problems. The focus group will be audiotaped and its content transcribed for qualitative analysis.

### Intervention 2 (experimental): Wiki

Group 2 participants will first be invited to participate in a Wiki. A Wiki is a collaborative writing web application used to create online content that anyone accessing it can edit or add to [21,22]. The Wiki was chosen as an innovative intervention that would allow participants to be involved in the CPG adaptation process in a place and at a rate best suited to their abilities as persons with TBI, without having to overcome transportation barriers. This could be especially interesting when CPGs are developed on a national basis and potential participants are located far from each other, such as in our case for Canadian rehabilitation services. Finally, there is increasing evidence of the applicability of electronic communication platforms for the population with TBI [23-26]. Indeed, approximately one person in two with TBI has access to the Internet [23,24] and around 60% of them use Facebook on a regular basis [27]. Thus, it is likely that if they are provided with specialized training material [24], the participants will be able to successfully participate in the guideline adaptation process.

Group 2 participants will receive an invitation by email, to include: 1) instructions about Wiki use, 2) the recommendation to be discussed online (the same one used by Group 1), and 3) a synthesis of the evidence that was used to produce that specific recommendation. The Wiki will be structured to describe the research project and present a synthesis of the evidence that was used to produce that specific recommendation. It will then allow the participants to express their opinion concerning the recommendation and specify their preferences about the recommendation, suggesting potential modifications to make to the recommendation. Support interventions will be provided electronically as needed by the moderator to encourage participants to interact on the Wiki. The participants will also receive prompts from the researchers two, four and six days after the initial email to encourage them to participate in the Wiki. They will have one week to do so.

One week after the end of the first data collection, in accordance with the crossover design, Group 1 participants will be assigned to the Wiki intervention and Group 2 participants will be assigned to the discussion group intervention. The procedure will be repeated with a second recommendation.

### Instruments and measures

Very few validated tools exist to measure the acceptability and feasibility of health interventions, and none were applicable to our trial. Therefore, we created tools based on solid conceptual frameworks and pre-tested them with patients from the population of interest.

### Acceptability

The acceptability of both methods for individuals with TBI will be assessed immediately after each trial, using electronic surveys. The questionnaire will be developed based on Sidani and Braden’s conceptual framework of accessibility [28]. In line with this framework, the questionnaire will document the participants’ perceptions with regard to the appropriateness of the identified method, its fit with usual habits, perceived effectiveness, perceived consequences and likelihood of re-using the method. For each aspect, the participants will be asked to rate their agreement with a statement using a 10-point visual analog scale ranging from 0 (I totally disagree with this sentence) to 10 (I totally agree with this sentence) [29]. They will also be asked to explain their answer using an open-ended qualitative question. After testing both methods (control and Wiki), the participants will be asked to answer a short open-ended questionnaire to document their preferences regarding the two methods. The questionnaires will be pre-tested with three individuals with TBI who would be eligible to, but would not participate in the study.

### Feasibility

The feasibility of the intervention will be evaluated using three indicators: a) the number of participants who attend the discussion group or access the Wiki, b) the number of participants who complete the intervention (attend the whole group or provide their opinion on the recommendations); and c) the number of support interventions required in the group and the Wiki [28]. A research professional using a pre-formatted template form will document the indicators.

### Effectiveness

The effectiveness of the two methods in producing useable recommendations will be evaluated by submitting the adapted recommendations to a panel of 20 potential users (individuals with TBI, clinicians, managers and policymakers). In this study effectiveness is defined as the capacity of a method to produce recommendations that are clear, accurate, appropriate and useful for users to guide their activities. The experts will be blinded to the methods used to adapt the recommendations. They will be recruited from the scientific committee that oversees the larger guideline adaptation process mentioned previously, and from community-based TBI associations similar to the one in which the individuals with TBI participating in the recommendations adaptations will be recruited. They will receive an email including the original recommendations, the recommendations produced by the participants, and a link to a questionnaire in which they will be invited to independently rate the clarity, accuracy, appropriateness and usefulness of each of the four recommendations (two conditions and two recommendations) using 10-point Likert scales [29]. They will also be invited to provide frank comments to justify their ratings.

### Analysis

Within-subject analysis using the Wilcoxon signed-rank test [30] will be performed to compare the acceptability score of the two methods. Feasibility indicators will be reported using descriptive statistics. The effectiveness of the methods as rated by the experts will be compared to the score provided by the experts for the perceived clarity, accuracy, appropriateness and usefulness of the recommendations, using generalized linear models (two methods and two recommendations) (Statistical Package for the Social Sciences (SPSS), Chicago, Illinois, United-States, using generalized estimating equations (GEE) since both independent variables are repeated measures). The selected distribution will depend on the distribution of the data. The statistical analysis will be performed using SPSS 20. All the qualitative data (discussion group and qualitative comments stemming from the questionnaires) will undergo a directed content analysis performed using NVivo 9.0 (QSR International, Burlington, Massachusetts, United-States.

## Discussion

This pilot trial will be the first to evaluate methods for involving individuals with TBI in CPG development. Given the severe impairments and/or disabilities experienced by individuals with TBI, it is likely that the optimal method could be used with success in other populations with disabilities, such as those living with stroke or spinal cord injury.

This pilot study has some limitations. Indeed, we have chosen a very pragmatic approach to test the two methods of adapting recommendations with a limited number of individuals with TBI. The pragmatic characteristic of our trial is important to test the feasibility of the two methods in a ‘real’ context of guideline adaptation, but it also raises issues. We have chosen not to control our sample for any type of disability (motor, cognitive or sensorial) in order to be representative of the population of individuals with moderate to severe TBI. Nor will we select our sample based on their ease with the use of the technology, so as to avoid eliciting an ‘expert’ group of patients not representative of the TBI population. Individuals with TBI often have disabilities that may influence their participation in the study, as well as their ability to conduct tasks as requested. For example, a participant might simply feel too tired and fall asleep in the middle of a focus group, limiting the interaction with other members of the group. The setting in which the study will take place (association’s office) is a familiar one for the participants, who sometimes visit it daily. This may make participants feel more comfortable and facilitate their involvement in the study. However, although participants will be instructed not to discuss their participation, contamination may occur during the trial. We will instruct the association employee to be sensitive to this issue and invite participants to share their thoughts with us.

Our trial will allow for the use of optimal IPP methods in a larger project of adapting guidelines for the rehabilitation of individuals with TBI. Ultimately the results will inform the science of CPG development and contribute to the growing knowledge about IPP in rehabilitation settings.

## Trial status

At the time of manuscript submission, the study team was preparing evaluation tools and planning recruitment.

## Abbreviations

CPG: Clinical practice guidelines; IPP: Involvement of patient and population; SPSS: Statistical Package for the Social Sciences; TBI: Traumatic brain injury.

## Competing interests

The authors declare that they have no competing interests.

## Authors’ contributions

MEL designed the study, wrote the protocol, obtained the funding and ethical approval, coordinated the study, led the protocol manuscript redaction and took the lead in writing the manuscript. KP helped coordinate the study, write the manuscript and contributed to the protocol manuscript redaction. MPG participated in the conception and coordination of the study, helped draft the manuscript and contributed to the protocol manuscript redaction. All authors read and approved the final manuscript.

## Supplementary Material

Additional file 1 **Recommendations to be adapted ([**[1]**], p38 and p43 respectively).** Click here for file
