# Supplementary material for: Evaluation of the acceptability, feasibility and effectiveness of two methods of involving patients with disability in developing clinical guidelines: study protocol of a randomized pragmatic pilot trial
Source: Trials. 2014 Apr 10;15:118. doi: 10.1186/1745-6215-15-118 (PMC4022363; doi:10.1186/1745-6215-15-118)
Supplement: Additional file 1 — Recommendations to be adapted ([[1]], p38 and p43 respectively). [file 1745-6215-15-118-S1.docx]

**Additional file 1**

**Recommendations to be adapted**

**Recommendation 1**: For optimal outcomes, higher intensity rehabilitation featuring early intervention should be delivered by specialist multidisciplinary teams.

(Scottish Intercollegiate Guideline Network: *Brain injury rehabilitation in adults.* Edinburgh: SIGN; 2013. p. 38)

**Recommendation 2**: Planned discharge from inpatient rehabilitation to home for patients who have experienced an ABI [Acquired Brain Injury] provides beneficial outcomes and should be an integrated part of treatment program.

(Scottish Intercollegiate Guideline Network: *Brain injury rehabilitation in adults.* Edinburgh: SIGN; 2013. p. 43)
